# Ethnic differences in longitudinal latent verbal profiles in the millennium cohort study*

**DOI:** 10.1093/eurpub/ckw184

**Published:** 2016-10-10

**Authors:** Afshin Zilanawala, Yvonne Kelly, Amanda Sacker

**Affiliations:** Epidemiology and Public Health, University College London, London, UK

## Abstract

**Background:** Development of verbal skills during early childhood and school age years is consequential for children’s educational achievement and adult outcomes. We examine ethnic differences in longitudinal latent verbal profiles and assess the contribution of family process and family resource factors to observed differences. **Methods:** Using data from the UK Millennium Cohort Study and the latent profile analysis, we estimate longitudinal latent verbal profiles using verbal skills measured 4 times from age 3–11 years. We investigate the odds of verbal profiles by ethnicity (reported in infancy), and the extent observed differences are mediated by the home learning environment, family routines, and psychosocial environment (measured at age 3). **Results:** Indian children were twice as likely (OR = 2.14, CI: 1.37–3.33) to be in the high achieving profile, compared to White children. Socioeconomic markers attenuated this advantage to nonsignificance. Pakistani and Bangladeshi children were significantly more likely to be in the low performing group (OR = 2.23, CI: 1.61–3.11; OR = 3.37, CI: 2.20–5.17, respectively). Socioeconomic and psychosocial factors had the strongest mediating influence on the association between lower achieving profiles and Pakistani children, whereas for Bangladeshi children, there was mediation by the home learning environment, family routines, and psychosocial factors. **Conclusion:** Family process and resource factors explain ethnic differences in longitudinal latent verbal profiles. Family resources explain verbal advantages for Indian children, whereas a range of home environment and socioeconomic factors explain disparities for Pakistani and Bangladeshi children. Future policy initiatives focused on reducing ethnic disparities in children’s development should consider supporting and enhancing family resources and processes.

## Introduction

Early childhood development is an influential predictor of later life academic and employment outcomes.^1^ The development of verbal capacities in the early years and school age years are consequential for children’s educational achievement, college completion, and adult labour force outcomes.^2^ Describing any observed differences in early childhood verbal abilities and the associated explanatory factors is important given the links between verbal skills in childhood and future life chances,^3^ and the economic benefits of intervening during early childhood to reduce long-term inequalities.^4^

Documenting and explaining ethnic patterning of verbal skills in early childhood is under-researched in the UK. One recent UK study using a repeat cross-sectional analysis found significant ethnic disparities (Indian, Pakistani, Bangladeshi, Black African and Black Caribbean groups) in verbal development in children up to 7 years of age^5^ and that differences between ethnic minority children and their White peers diminished with increasing age. Empirical work has not used a longitudinal perspective when examining ethnic disparities in verbal skills and heterogeneity in verbal development has not been considered.^5^ Methods beyond simple mean differences at single time points, such as growth trajectories or latent profile analyses,^6^ have not been employed on data from the UK.

Achievement gaps between ethnic minority and white children have been examined in the US. Latin American origin children performed less well on average, on assessments of expressive language and reading abilities whereas children of Asian origin outperformed their White peers in reading abilities.^7^^,^ ^8^ Comparing verbal assessments among 4–5 year olds in four OECD countries, ethnic minority children performed more poorly compared with children from the ethnic majority.^9^ Longitudinal data in the US suggest different patterns of verbal development by race/ethnic group and immigrant status;^10^^,^^11^ Black American, Mexican American, and Puerto Rican school aged children had deficits in initial levels and declines in verbal abilities over time relative to White peers. However, more favourable verbal trajectories, including improving verbal performance from initial deficits, were documented for immigrant children within race/ethnic groups.^10^ Similar favourable verbal growth patterns have been shown for Latin American children, thus narrowing the gap with White children over childhood.^11^

Family resources, which include family socioeconomic position, often proxied by family income^12^ and language spoken at home, are strongly associated with academic achievement for children.^9^ Family process factors, such as the home learning environment, warm and supportive parenting, family routines, and maternal mental health, have also been linked to academic performance and favourable developmental outcomes.^13^

In this study, we consider the heterogeneity in children’s verbal scores over time by using a latent profile analysis and four waves of data from the Millennium Cohort Study. We examine^1^ what longitudinal latent verbal profiles are observed;^2^ what are the ethnic differences in longitudinal latent verbal profiles; and^3^ what is the contribution of the home learning environment, family routines, and psychosocial environment in explaining observed ethnic differences in longitudinal latent verbal profiles.

## Methods

### Millennium Cohort Study

The Millennium Cohort Study (MCS) is a nationally representative longitudinal study of infants born in the UK in 2000–2002.^14^^,^^15^ The MCS sample was clustered within electoral wards. Disadvantaged residential areas and areas with a high proportion of ethnic minority people are over-represented. Parents were interviewed when cohort members were approximately 9 months old, 3, 5,7, and11 years. Trained interviewers carried out cognitive assessments at ages 3, 5, 7, and 11.

### Verbal skills

Verbal skills were assessed using a subset of the British Ability Scales II (BAS II), which is a battery of cognitive abilities and educational achievement tests suitable for use from ages 2 years 6 months to 17 years 11 months.^16^ The individual subscales are widely validated, age appropriate, can be analysed separately, and have been shown to predict later child cognitive performance.^17^ Data were available on the BAS II Naming Vocabulary Subscale (age 3 and 5 years) which measures vocabulary and expressive reasoning, the Word Reading subscale (age 7 years) involving verbal reasoning, and the Verbal Similarities subscale (age 11) assessing children’s verbal reasoning and verbal knowledge^18^ The subscale scores used in this study are standardized to mean 50 and standard deviation 10 and are adjusted for both item difficulty and age.

### Ethnicity

Ethnic categories for analysis were: White, Indian, Pakistani, Bangladeshi, Black Caribbean, Black African, and other. The ‘other’ group includes mixed ethnic groups and ethnic minority groups that could not be categorized into any of the otherwise defined groups.

### Explanatory factors

All variables were assessed at 3 years unless noted otherwise. Demographic controls were whether or not the cohort member was a firstborn and mother’s age at the time of birth. Socioeconomic and home environment variables are considered as mediators. Socioeconomic markers were equivalised family income in quintiles and whether the primary household language was English or another language. Three domains of the home environment were measured at age 3: learning, family routines, and psychosocial environmental factors. Home learning environment measures were: parental basic skills difficulties (9 month data); and frequency of learning activities: someone reads stories to the child, visits to the library, help with alphabet, numbers/counting, learning songs, poems and rhymes, and does drawing and painting. Family routines were whether the child had regular bedtimes and mealtimes. Markers of the psychosocial environment were: maternal psychological distress,^19^ parent-child relationship,^20^ discipline strategies—a composite score (α = 0.71) of seven items from the Conflict Tactics Scale,^21^ nine parent-child items from the Home Observation for Measurement of the Environment (HOME) Inventory,^22^ whether the mother felt she was a competent parent, whether the family had lots of rules, and whether these rules were enforced.

### Sample

Child verbal skills are moderated by multiple births and therefore we analysed data on singleton-born cohort members with observed ethnicity and who had at least one verbal assessment across the four sweeps.^23^ The analytic sample was 16 704 after multiply imputing missing values on explanatory factors and verbal assessments. We applied Multiple Imputation by Chained Equations (MICE) techniques and imputed 25 datasets.^24^ Further information on the imputation model and missingness in analysis variables is in Appendix A of the online Supplementary Material.

### Analytical approach

To identify and characterize longitudinal latent verbal profiles, we used a three-step latent profile analysis (LPA).^25^ Further detail on the methodology can be found in Appendix B, online Supplementary Material. In the base model (Model 0) we present estimates of ethnic differences. Then we separately adjusted for 5 sets of controls and mediators: Model 1: demographic controls; Model 2: socio-economic characteristics; Model 3: home learning environment; Model 4: family routines; Model 5: psychosocial environment; and Model 6: simultaneously adjusts for all covariates. We use multinomial regression models and present odds ratios. All analyses accounted for sample design and non-response.

## Results

**Figure 1:**
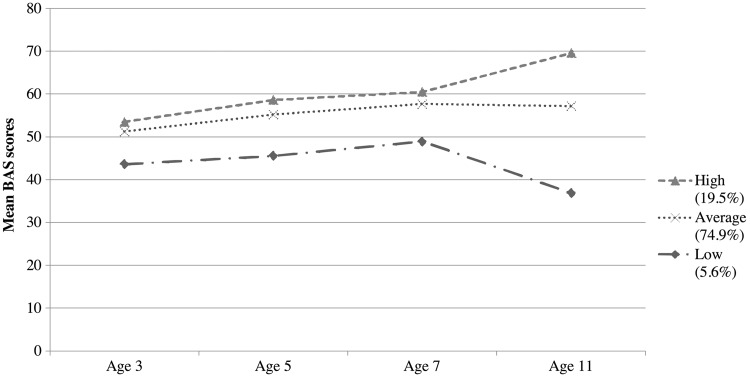
Longitudinal latent verbal profiles Note: BAS, British Ability Scales

LPA revealed the optimal solution to be three longitudinal latent verbal profiles. Fit indices are presented in table 1. Models beyond five profiles were contraindicated by the fit indices. The additional one or two profiles beyond a three-profile solution reflected variants of low and average verbal performances, did not offer distinct substantive insight related to verbal performance, and lastly these additional profiles were small, with prevalence below 5%. These verbal profiles are depicted in figure 1. The largest group was named the “average” (74.9% of the sample). The scores of this group at each age of assessment were closer to the overall sample mean, with mean scores ranging between 51 and 57 across the four assessment periods. In contrast to this group, a “low” group (5.6% of the sample) had the poorest verbal performance across childhood, with mean scores ranging from 37 to 44. In contrast to these two groups, the “high” (19.5% of the sample) group included children with the highest verbal scores, with means ranging from 54 to 70 across the four assessment points.

**Table 1:** Fit indices for latent profile analyses (*N* = 16 704)

	Number of profiles			
	2	3	4	5
Log-likelihood	−276,535	−**276,390**	−276,303	−276,259
BIC	487,280	**487,032**	486,942	487,032
BIC adj.	487,194	**486,931**	486,824	486,784
AIC	487,071	**486,785**	486,656	486,785
Entropy	0.91	**0.73**	0.72	0.66

Notes: BIC = Bayesian information criterion, a measure of model fit; smaller values indicated better fit; BIC adj. = BIC adjusted for sample size; smaller values again indicate better fit; Entropy = measure of the accuracy of classification of children in latent profiles and of profile differentiation; higher values indicate better classification; AIC = Akaike information criterion, a measure of model fit; smaller sizes indicate better fit. Bolded column indicates the profile solution retained for subsequent modelling. All analyses are weighted with MCS overall weights. Sample sizes are limited to those who have observations on ethnicity.

Table 2 shows the mean scores on verbal assessments used in our LPA by cohort member’s ethnicity across four sweeps, with White children as the reference group. At age 3, children from ethnic minority groups had significantly lower verbal skills scores, on average, compared with their White peers. Pakistani and Bangladeshi children had the lowest verbal scores with scores more than a standard deviation below the mean (36.2 and 33.8, respectively). The ethnic minority disadvantage remained at age 5. At age 7, Pakistani, Bangladeshi, and Black Caribbean children no longer differed from White children on their verbal scores, and Indian and Black African children performed significantly better than White children, with scores nearly 1 standard deviation above the mean. At age 11, Indian children continued to have an advantage in verbal scores. Although Pakistani and Bangladeshi children scored significantly lower on verbal tests than White children, their average scores were above the standardized sample mean. At age 11 there were no differences in verbal scores between White and Black Caribbean and Black African children. Appendix C (online Supplementary Material), table A1 illustrates explanatory factors by ethnicity. Socioeconomic disadvantage was associated with ethnic minority groups. There was considerable heterogeneity in the distribution of the home learning environment, family routines and psychosocial factors by ethnicity.

**Table 2:** Cross-sectional mean scores on BAS tests by ethnicity using T-Scores

	BAS Naming Vocabulary		BAS Word Reading	BAS Verbal Similarities
	Age 3	Age 5	Age 7	Age 11
Overall mean	50.0	54.7	56.7	58.3
Ethnicity				
White	51.1	55.8	56.6	58.4
Indian	43.8***	51.2***	60.8***	61.5***
Pakistani	36.2***	41.9***	56.6	54.9**
Bangladeshi	33.8***	41.2***	58.3	52.4***
Black Caribbean	47.0***	51.7***	55.3	57.6
Black African	42.0***	46.4***	58.2**	58.9
Other	44.8***	49.0***	57.2	58.9
*N*	14,198	14,675	13,136	12,720

Notes: All tests are standardized to a mean of 50 and standard deviation of 10. All means are weighted by sample weights at year of assessment. Multiple births are excluded. Means are adjusted for age and gender at sweep. BAS, British Ability Scales.

*** *P* < 0.001.

** *P* < 0.01.

* *P* < 0.05.

Table 3 illustrates the odds of having high and low performing profiles by ethnic group. The reference category is average performing. The first panel presents results for the highest performing group compared to the average performing profile. In Model 0, Indian children, as compared to White children, were significantly more likely to be in the high achieving profile (odds ratio, OR = 2.14, confidence interval, CI = 1.37–3.33). Adjustment for demographic controls did not influence this estimate (Model 1) whereas socio-economic characteristics attenuated the difference to non-significant levels (Model 2). Adjustment for markers of home learning, family routines, and psychosocial environment amplified the association for Indian children (Models 3–5). In fully adjusted models, the higher odds for Indian children were no longer statistically significant but were of meaningful magnitude (OR = 1.77, CI = 0.94–3.32).

**Table 3:** Odds Ratios (95% CI) from Multinomial Logistic Regressions Predicting Longitudinal Latent Verbal Profiles by Ethnicity^1^ (*N* = 16,704)

	Model 0	Model 1	Model 2	Model 3	Model 4	Model 5	Model 6
**High**							
Indian	2.14**	2.20**	1.56	2.52***	2.27***	2.40***	1.77
	(1.37, 3.33)	(1.39, 3.49)	(0.83, 2.91)	(1.56, 4.08)	(1.45, 3.56)	(1.51, 3.82)	(0.94, 3.32)
Pakistani	0.82	0.98	0.81	1.01	0.87	1.07	0.98
	(0.51, 1.29)	(0.61, 1.56)	(0.44, 1.5)	(0.62, 1.65)	(0.54, 1.4)	(0.66, 1.74)	(0.51, 1.86)
Bangladeshi	0.51	0.66	0.55	0.77	0.54	0.75	0.75
	(0.17, 1.46)	(0.22, 2.01)	(0.19, 1.59)	(0.28, 2.1)	(0.18, 1.58)	(0.27, 2.06)	(0.27, 2.11)
Black Caribbean	0.86	0.85	1.06	0.92	0.95	0.89	1.04
	(0.46, 1.62)	(0.44, 1.63)	(0.56, 2.02)	(0.48, 1.77)	(0.51, 1.77)	(0.46, 1.72)	(0.53, 2.04)
Black African	1.32	1.26	1.24	1.72*	1.52	1.52	1.39
	(0.80, 2.17)	(0.74, 2.14)	(0.7, 2.2)	(1.01, 2.94)	(0.92, 2.53)	(0.91, 2.55)	(0.77, 2.51)
Other	1.29	1.20	1.10	1.46	1.37	1.47	1.14
	(0.78, 2.13)	(0.72, 2.01)	(0.6, 2)	(0.88, 2.42)	(0.83, 2.27)	(0.87, 2.49)	(0.62, 2.12)
**Low**							
Indian	0.47	0.43	0.52	0.38*	0.42	0.37*	0.47
	(0.18, 1.21)	(0.16, 1.17)	(0.19, 1.46)	(0.14, 0.99)	(0.17, 1.06)	(0.15, 0.94)	(0.16, 1.38)
Pakistani	2.23***	1.86***	1.50	1.53*	1.80**	1.36	1.26
	(1.61, 3.11)	(1.33, 2.61)	(0.84, 2.66)	(1.07, 2.2)	(1.27, 2.54)	(0.96, 1.93)	(0.67, 2.34)
Bangladeshi	3.37***	2.71***	2.25*	2.15**	2.90***	1.95**	1.75
	(2.20, 5.17)	(1.76, 4.17)	(1.15, 4.37)	(1.34, 3.44)	(1.86, 4.52)	(1.20, 3.16)	(0.84, 3.65)
Black Caribbean	1.32	1.30	0.95	1.20	1.10	1.23	0.96
	(0.75, 2.33)	(0.74, 2.29)	(0.53, 1.71)	(0.68, 2.12)	(0.62, 1.93)	(0.69, 2.18)	(0.54, 1.7)
Black African	0.83	0.87	0.65	0.60	0.62	0.63	0.54
	(0.40, 1.69)	(0.42, 1.79)	(0.29, 1.44)	(0.29, 1.21)	(0.3, 1.28)	(0.31, 1.28)	(0.23, 1.26)
Other	0.56	0.58	0.51	0.51	0.48	0.45	0.48
	(0.22, 1.14)	(0.23, 1.19)	(0.2, 1.13)	(0.21, 1.03)	(0.19, 1.001)	(0.19, 0.93)	(0.18, 1.11)

*** *P* < 0.001.

** *P* < 0.01.

* *P* < 0.05.

Notes: All estimates are weighted with analytic weights.

1 White is the reference group for ethnicity and average performing is the reference latent profile.

Model 0 (M0): Ethnicity; Model 1: M0 + Demographic controls; Model 2: M0 + Socioeconomic; Model 3: M0 + Home learning; Model 4: M0 + Family routines; Model 5: M0 + Psychosocial; Model 6: Fully adjusted model.

The second panel of table 3 shows the odds of being in the low performing profile compared to being in the average profile. In the unadjusted model, as compared to White children, Pakistani and Bangladeshi children were significantly more likely to be in the low performing group (OR = 2.23, CI = 1.61–3.11; OR = 3.37, CI = 2.20–5.17, respectively). Adjustment for demographic confounders attenuated differences for Pakistani and Bangladeshi children (Model 1). Adjustment for socio-economic factors attenuated the difference between Pakistani and White children to non-significant levels, but did not completely explain the differences for Bangladeshi children (Model 2). Both home learning and family routines partially explained the highest odds for these two groups (Models 3 and 4). Adjusting for psychosocial factors explained the highest odds for Pakistani children and attenuated the odds for Bangladeshi children (Model 5). In fully adjusted models, the highest odds for Pakistani and Bangladeshi children were no longer apparent (OR = 1.26, CI = 0.67–2.34; OR = 1.75, CI = 0.84–3.65, respectively).

We separately adjusted for individual markers of the psychosocial environment (Appendix C, table A2, online Supplementary Material). Each marker explained 1–24% of the higher odds of being in the low achieving profile for Pakistani and Bangladeshi children, with the HOME inventory reducing estimates the most (OR = 1.84, CI = 1.32, 2.56; OR = 2.60, CI = 1.66–4.07, respectively).

Black Caribbean and Black African children were no more or less likely than White children to be in either the high or low achieving profiles.

## Discussion

Our analyses found three longitudinal verbal latent profiles: low (5.6%), average (74.5%), and high (19.5%) performing groups. Indian children were twice as likely (unadjusted estimates) as White children to be in the high achieving profile and socioeconomic and demographic markers, as operationalized here, explained this advantage. Pakistani and Bangladeshi children in unadjusted models were significantly more likely to be in the low achieving profile than their White peers. Socioeconomic and psychosocial disadvantage did most to explain the lower achieving profiles of Pakistani children, while a range of markers of ethnic disadvantage—socioeconomic, home learning, family routines, and psychosocial mediators—explained the observed inequality in low verbal scores for Bangladeshi children. We found no differences in the longitudinal latent verbal profiles between White and Black Caribbean and Black African children.

Investigating verbal profiles during early childhood using a data-driven process has revealed verbal profiles similar to our findings.^26^ This research has underscored the importance of latent profiles to highlight heterogeneity in verbal performance, particularly when examining ethnic disparities.^6^ A recent study using the MCS similarly finds different verbal performance profiles and emphasizes the use of multiple assessments of verbal performance to avoid regression to the mean, a potential pitfall of only using one measurement occasion.^27^

High and low longitudinal latent verbal profiles were associated with minority status, but the factors that explain the ethnic patterning appear to be different. Sociodemographic measures explained the high achieving profiles of Indian children who on average grow up in economically advantaged families and have mothers who are primarily English proficient.^28^ Family sociodemographic measures and parental English proficiency are key factors in children’s verbal skills development.^29^^,^^30^ Conversely, the low achieving profile of Pakistani children was also accounted for after adjusting for sociodemographic measures. Two-thirds of Pakistani families are in the bottom income tertile^31^ and mothers of Pakistani children have one of the lowest rates of English proficiency.^28^ Our results underscore the importance of exposure to an English-speaking home environment, not just for verbal skills acquisition but because parents who are English proficient can negotiate and navigate UK schools and social institutions to their children’s benefit.

A combination of socioeconomic, home learning, family routines, and psychosocial mediators explains the low performing profiles of Bangladeshi children. Evidence suggests warm caring home environments and favourable psychosocial contexts interacting with family routines, such as regular bed and meal times and story reading, are beneficial for child development.^32^^,^^33^ Markers of the psychosocial environment attenuated the higher odds of low performance for Pakistani children to nonsignificance and for Bangladeshi children by 45%. Each marker of the psychosocial context reduced the higher odds by 1–24%. The HOME inventory, maternal psychological distress, and parent-child relationship (Pianta scale) each attenuated odds by 16-24%. Nurturance, discipline, and language use, all of which can be are encapsulated in the HOME inventory and the Pianta scale, are linked to reducing school readiness gaps.^34^ Verbal performance was also sensitive to maternal psychological distress, supporting evidence of higher prevalence of maternal distress among mothers of ethnic minority children^35^ and the deleterious effects of mental distress on children’s verbal skills.^36^ Thus, socioeconomic factors are not always sufficient to explain the variation in children’s longitudinal verbal performance. Indeed, the family stress model suggests that financial hardship can disrupt parents’ socioemotional resources and compromise parent-child interactions,^37^ which may in turn affect child verbal performance.

Two other UK studies examine differences in verbal abilities by ethnicity or migration status. A cross-national study examining differences in verbal skills by maternal migration status reported that 4–5 year old children of immigrants perform more poorly than children of natives.^9^ This study aggregated all ethnic minority children in a catchall group of ‘children of foreign-born parents’, making it difficult to compare with our results. Using a detailed ethnic classification revealed variations in the odds of high performing, low performing, or similar performance profiles compared with White peers. Our results compare to the aforementioned study in finding adverse verbal performance for children growing up in families in which English is not the primary spoken language.^9^ Another study, using the MCS and more detailed ethnic categories, revealed verbal disadvantages at ages 3, 5, and 7 for all ethnic minorities as operationalized in our study.^5^ However, this study investigated repeated cross-sectional means, which may obscure patterns of verbal achievement across early childhood. The authors also found verbal disadvantages at ages 3, 5 and 7 for Black Caribbean children and at ages 3 and 5 for Black African children (unadjusted estimates), whereas our results indicate no disparities in verbal performance between Black Caribbean and African children and the White majority group from ages 3 to 11. The same study finds verbal disadvantages for Indian children (ages 3 and 5), and our results highlight a positive profile for Indian children. These differences between the previous study and our findings may very well be attributable to the design approaches; a virtue of using a longitudinal design, such as LPA, is revealing stable profiles across time instead of relying on mean differences at one point in time. Highlighting such variation in developmental pathways has been significant in other areas of child development, for example child behaviour and substance use, leading to more effective intervention strategies.^38^

Our findings on Black Caribbean and Black African children lie in stark contrast to previous findings.^5^ Although ethnic minority status has been linked to disadvantages in child health and developmental,^35^ the distribution of parental sociodemographic and economic factors differ between ethnic minority groups. For example, Black Caribbean children are raised in families in which the primary language spoken at home is English and this exposure to an English-speaking environment has been associated with better performance on standardized tests.^7^ Black Caribbean families have a long migration history to the UK and the vast majority of mothers of Black Caribbean children are UK born. Successive generations following migration are associated with upward economic mobility,^5^ which in turn is predictive of child cognitive development. That we do not find disadvantages for Black African children is not surprising as there is evidence of health advantages for Black African children and adolescents.^39^ Over one-half of mothers of Black African children have at least NVQ4 education (first degree/diploma or higher) and nearly one-third of Black African mothers are employed full-time, and these markers are correlated with child health and development.^7^

A virtue of this study is that we examined data on objective measures of verbal ability in children. Secondly, we took advantage of the longitudinal nature of the data to capture the heterogeneity of children’s verbal skills. Despite the rich information on family processes and resources, it could be the case that we have underestimated the effects if socioeconomic measures and family environment variables lack precision, as they are proxies for a myriad of ill-defined socio-environmental factors.^13^ It is undoubtedly possible that children may experience differential treatment by teachers and such classroom-based racism may influence variation in verbal scores. Data constraints limited our ability to control for unmeasured characteristics, which may be correlated to both ethnicity and verbal development, for example, parental motivation, personality, and educational beliefs.

Children’s verbal skills are consequential to their future life chances. Our work found both family process and resource factors mediated ethnic differences in longitudinal latent verbal profiles. Given the economic and long-term benefits to early childhood investments, policy interventions can be developed to support and enhance family resources and processes to reduce ethnic inequalities in children’s development. For example, successful interventions alongside welfare reforms have focused on helping parents promote child development and verbal skills in the home and directly teaching socially and economically disadvantaged children.^40^ It is essential for policies to continue to develop to close the gap in ethnic inequalities in verbal skills.

## Supplementary data

Supplementary data are available at *EURPUB* online.

Supplementary Data
