# Supplementary material for: Ethnic differences in longitudinal latent verbal profiles in the millennium cohort study*
Source: Eur J Public Health. 2016 Oct 10;26(6):1011–6. doi: 10.1093/eurpub/ckw184 (PMC5172493; doi:10.1093/eurpub/ckw184)
Supplement: Supplementary Data [file 988e022d43bc645193206975f1954c09_ejph-2016-05-om-0370-File003.docx]

**Supplementary Material**

*Appendix A. Latent Profile Analysis*

Latent profile analysis (LPA)(1) is a person-centered approach that is used to identify patterns of verbal development across the four measurement points. A person-centered approach emphasizes patterns among individuals as opposed to variables, and clusters individuals into subgroups (latent profiles) with different probability distributions(2).

In the first step, an optimal latent profile solution was determined by estimating a series of latent profile models using the 4 assessments of child verbal scores, incrementing the number of profiles in successive models, and comparing models based on conceptual, empirical, and practical considerations in an iterative manner(3). Model comparison used standard model fit indices, which include the Bayesian information criterion(4) (BIC); the sample-size-adjusted BIC(5); ; the Akaike information criterion(6) (AIC), lower scores indicate better fitting models; the Lo–Mendell–Rubin (LMR) likelihood ratio test (not available for the imputed analyses), which tests the estimated model against a model reduced by one profile; and entropy, a higher score of which reflects greater accuracy in classification. We adjusted for child age and gender to control for their influence on classification. A three-profile solution was derived using conceptual and statistical evidence.

After finding the optimal profile solution, we implemented the R3STEP method in Mplus (version 7.11) which accounts for measurement error (or uncertainty in profile membership) in the most likely profile variable(7). Disregarding the uncertainty in profile membership can increase bias and decrease precision when conducting subsequent multinomial logistic regressions and estimating magnitudes of the associations between latent profiles and covariates(8).Thus, in the second step the most likely profile membership and measurement error were obtained. In the third step, we use the most likely profile variable to assess ethnic differences in longitudinal latent verbal profiles and the contribution of the home learning environment, family routines, and psychosocial environment in explaining observed differences in verbal skills while accounting for measurement error in classification. Importantly, the three-step procedure ensures stable latent profile assignments when examining sequences of models with covariate adjustment(8). Mplus reports logit coefficients which were converted to odds ratios.

*Appendix B. Multiple imputation*

The analytic sample was 16,704 after multiply imputing missing values on explanatory factors and verbal assessments. The percent of missingness was between 0 and 21 percent for explanatory factors, of which parent-child relationship, discipline strategies, and whether the mother felt she was a competent parent had the highest percent of missingness (Table B2). Missing information on verbal scores was between 12 and 24 percent.

The imputation model included all explanatory factors, auxiliary variables measuring socio-demographic characteristics from previous interviews, and design variables to consider the clustered nature of the data. Using STATA, we applied Multiple Imputation by Chained Equations (MICE) techniques which account for uncertainty about missing values by imputing several values for each missing data point (9). We imputed 25 datasets, consolidated results from all imputations using Rubin’s combination rules (10), and excluded cases with imputed values on ethnicity.

Post-imputation diagnostics did not reveal large deviations in latent profile solutions and estimates on verbal skills and explanatory factors between full information maximum-likelihood (FIML) analyses and MI analyses (Analyses not shown but available upon request). Imputation literature recommends using imputation on all variables when the imputation model includes auxiliary variables, as it does in our analyses, because such variables provide extra information on the outcome variable(11).

| **Table B2. Percent Missingness for Verbal Scores and All Explanatory Factors (in percent)** | |
| --- | --- |
| Verbal scores |  |
| Age 3 | 15.0 |
| Age 5 | 12.1 |
| Age 7 | 21.4 |
| Age 11 | 23.9 |
| Ethnicity | 0.0 |
| Equivalised household income | 11.0 |
| Parent-child relationship | 20.9 |
| Home language | 10.8 |
| Mother's age at birth | 3.0 |
| Child is first born | 2.9 |
| Parental basic skills difficulties | 3.1 |
| Someone reads stories to child | 10.8 |
| Visits to the library | 10.8 |
| Help with the alphabet | 10.8 |
| Help with numbers/counting | 10.8 |
| Learning songs, poems or rhymes | 10.8 |
| Does drawing and painting | 10.8 |
| Regular bedtimes | 10.8 |
| Regular mealtimes | 10.8 |
| Mother's psychological distress | 20.0 |
| Discipline strategies | 20.6 |
| HOME Inventory | 16.0 |
| Maternal parenting competence | 20.7 |
| Family rules | 10.8 |
| Enforcement of rules | 10.8 |
| Notes: Figures are based on 16,704 children. Sample is exclusive to cohort members with at least one verbal assessment across 4 sweeps and observed ethnicity but excludes multiple births. | |

References

1. Gibson WA. Three multivariate models: Factor analysis, latent structure analysis, and latent profile analysis. Psychometrika. 1959;24(3):229-52.

2. Lubke GH, Muthén B. Investigating population heterogeneity with factor mixture models. Psychological methods. 2005;10(1):21.

3. Nylund KL, Asparouhov T, Muthén BO. Deciding on the number of classes in latent class analysis and growth mixture modeling: A Monte Carlo simulation study. Structural equation modeling. 2007;14(4):535-69.

4. Schwarz G. Estimating the dimension of a model. The annals of statistics. 1978;6(2):461-4.

5. Sclove SL. Application of model-selection criteria to some problems in multivariate analysis. Psychometrika. 1987;52(3):333-43.

6. Akaike H. Factor analysis and AIC. Psychometrika. 1987;52(3):317-32.

7. Asparouhov T, Muthén B. Auxiliary Variables in Mixture Modeling: Three-Step Approaches Using M plus. Structural Equation Modeling: A Multidisciplinary Journal. 2014;21(3):329-41.

8. Vermunt JK. Latent class modeling with covariates: Two improved three-step approaches. Political analysis. 2010;18(4):450-69.

9. Allison PD. Missing data: Quantitative applications in the social sciences. British Journal of Mathematical and Statistical Psychology. 2002;55(1):193-6.

10. Rubin DB. Multiple imputation for nonresponse in surveys: Wiley. com; 2009.

11. White IR, Royston P, Wood AM. Multiple imputation using chained equations: Issues and guidance for practice. Statistics in medicine. 2011;30(4):377-99.

*Appendix C.*

| **Table C1. Distribution of socioeconomic and demographic characteristics, home learning, family routines and psychosocial environment by ethnicity (Age 3)** | | | | | | | |
| --- | --- | --- | --- | --- | --- | --- | --- |
|  | White | Indian | Pakistani | Bangladeshi | Black Caribbean | Black African | Other |
| **Socioeconomic and demographic** |  |  |  |  |  |  |  |
| Equivalized household income |  |  |  |  |  |  |  |
| Lowest quintile | 16.0 | 16.1 | 45.8 | 53.7 | 36.8 | 36.1 | 21.2 |
| Second quintile | 17.9 | 18.6 | 33.7 | 27.9 | 20.7 | 20.0 | 21.5 |
| Third quintile | 20.9 | 20.0 | 10.9 | 8.5 | 14.7 | 14.2 | 19.6 |
| Fourth quintile | 22.5 | 16.8 | 4.9 | 6.5 | 17.2 | 13.1 | 16.9 |
| Highest quintile | 22.8 | 28.5 | 4.7 | 3.5 | 10.7 | 16.6 | 20.8 |
| Child is first born | 42.9 | 43.8 | 34.1 | 25.5 | 43.7 | 34.8 | 41.7 |
| Language spoken at home is primarily English | 97.4 | 18.3 | 7.2 | 2.0 | 96.5 | 41.1 | 45.8 |
| Mother's age at birth, years | 29.0 | 28.9 | 26.7 | 26.4 | 28.8 | 30.5 | 30.1 |
| **Home learning** |  |  |  |  |  |  |  |
| Parental basic skills difficulties (0-6) | 0.3 | 0.4 | 0.6 | 0.7 | 0.2 | 0.4 | 0.3 |
| Someone reads stories to child |  |  |  |  |  |  |  |
| Everyday | 63.1 | 47.9 | 35.6 | 26.4 | 50.2 | 33.8 | 52.6 |
| 1 to 6 days per week | 31.6 | 40.5 | 43.0 | 51.6 | 40.5 | 51.8 | 36.9 |
| Less often | 5.3 | 11.6 | 21.4 | 22.0 | 9.4 | 14.4 | 10.5 |
| Visits to the library |  |  |  |  |  |  |  |
| At least weekly | 7.5 | 7.5 | 8.4 | 5.4 | 8.5 | 8.7 | 9.6 |
| Less often | 92.5 | 92.5 | 91.6 | 94.6 | 91.5 | 91.3 | 90.4 |
| Help with the alphabet |  |  |  |  |  |  |  |
| At least weekly | 68.1 | 79.1 | 70.9 | 63.8 | 75.2 | 75.6 | 70.5 |
| Less often | 31.9 | 20.9 | 29.1 | 36.2 | 24.8 | 24.4 | 29.5 |
| Help with numbers/counting |  |  |  |  |  |  |  |
| At least weekly | 92.5 | 92.3 | 87.0 | 73.3 | 90.9 | 85.1 | 87.4 |
| Less often | 7.5 | 7.7 | 13.0 | 26.7 | 9.1 | 14.9 | 12.6 |
| Learning songs, poems or rhymes |  |  |  |  |  |  |  |
| At least weekly | 93.4 | 90.2 | 75.2 | 66.5 | 92.7 | 78.9 | 84.6 |
| Less often | 6.6 | 9.8 | 24.8 | 33.5 | 7.3 | 21.1 | 15.4 |
| Does drawing and painting |  |  |  |  |  |  |  |
| At least weekly | 94.8 | 95.1 | 90.2 | 88.4 | 92.0 | 86.7 | 92.6 |
| Less often | 5.2 | 4.9 | 9.8 | 11.6 | 8.0 | 13.3 | 7.4 |
| **Family routines** |  |  |  |  |  |  |  |
| Regular bedtimes |  |  |  |  |  |  |  |
| Usually/Always | 81.8 | 74.1 | 66.8 | 69.8 | 67.9 | 59.7 | 72.3 |
| Rarely/never | 18.2 | 25.9 | 33.2 | 30.2 | 32.1 | 40.3 | 27.7 |
| Regular mealtimes |  |  |  |  |  |  |  |
| Usually/Always | 92.7 | 88.0 | 79.3 | 80.1 | 82.8 | 77.6 | 87.0 |
| Rarely/never | 7.3 | 12.0 | 20.7 | 19.9 | 17.2 | 22.4 | 13.0 |
| **Psychosocial environment** |  |  |  |  |  |  |  |
| Mother's psychological distress, (0-24) | 3.2 | 4.2 | 5.8 | 6.3 | 3.8 | 4.4 | 4.2 |
| Parent-child relationship (30-75) | 64.0 | 64.1 | 61.1 | 59.7 | 63.9 | 63.6 | 63.3 |
| Discipline strategies (0-28) | 13.0 | 11.8 | 11.7 | 11.7 | 12.4 | 11.5 | 11.6 |
| HOME Inventory (0-1) | 0.1 | 0.1 | 0.2 | 0.2 | 0.1 | 0.1 | 0.1 |
| Maternal parenting competence |  |  |  |  |  |  |  |
| Better than average | 57.7 | 62.1 | 61.6 | 75.1 | 54.0 | 64.7 | 61.4 |
| Average or worse | 42.3 | 37.9 | 38.4 | 24.9 | 46.0 | 35.3 | 38.6 |
| Family rules |  |  |  |  |  |  |  |
| Lots of rules | 31.0 | 26.1 | 22.7 | 17.3 | 36.8 | 36.1 | 27.3 |
| Not many rules | 41.9 | 46.1 | 56.7 | 60.5 | 34.8 | 43.9 | 48.6 |
| It varies | 27.1 | 27.9 | 20.6 | 22.2 | 28.4 | 20.1 | 24.1 |
| Enforcement of rules |  |  |  |  |  |  |  |
| Strictly enforced | 50.5 | 42.9 | 28.1 | 28.8 | 54.0 | 45.4 | 41.9 |
| Not very strictly enforced | 22.6 | 33.6 | 48.8 | 50.0 | 22.4 | 26.0 | 30.4 |
| It varies | 26.9 | 23.4 | 23.1 | 21.2 | 23.5 | 28.6 | 27.6 |
| **N** | 13,778 | 475 | 847 | 344 | 415 | 427 | 418 |
| Notes: All means are weighted with sample weights. Discipline strategies and maternal psychological distress are coded as higher scores indicating more problems. Parent-child relationship and HOME inventory scores are coded as higher scores indicating more favorable circumstances. Sample is exclusive to cohort members with at least one verbal assessment across 4 sweeps and observed ethnicity, but excludes multiple births. Sample sizes are unweighted. | | | | | | | |

| **Table C2. Psychosocial factors for Pakistani and Bangladeshi children in low achieving profiles [Odds Ratios (95% CI)]** | | | | | | | | |
| --- | --- | --- | --- | --- | --- | --- | --- | --- |
|  | Model 0 (M0): Ethnicity | Maternal psychological distress | Parent-child relationship | Discipline strategies | HOME Inventory | Maternal parenting competence | Family rules | Enforcement of rules |
|  |  |  |  |  |  |  |  |  |
| **Low** |  |  |  |  |  |  |  |  |
| Pakistani | 2.23*** | 1.89 | 1.93 | 2.20 | 1.84 | 2.27 | 2.13 | 1.94 |
|  | (1.61 , 3.11) | (1.35 , 2.63) | (1.38 , 2.7) | (1.58 , 3.07) | (1.32 , 2.56) | (1.63 , 3.16) | (1.52 , 2.97) | (1.39 , 2.72) |
| % Attenuation from M0 |  | 21.0% | 18.0% | 1.9% | 24.4% | -2.0% | 6.2% | 17.3% |
| Bangladeshi | 3.37*** | 2.78 | 2.79 | 3.33 | 2.60 | 3.65 | 3.17 | 2.97 |
|  | (2.20 , 5.17) | (1.8 , 4.31) | (1.78 , 4.36) | (2.17 , 5.12) | (1.66 , 4.07) | (2.37 , 5.62) | (2.07 , 4.84) | (1.93 , 4.56) |
| % Attenuation from M0 |  | 15.8% | 15.6% | 1.1% | 21.5% | -6.4% | 5.2% | 10.4% |
| ***p<0.001 **p<0.01 *p<0.05 | | | | | | | | |
| Notes: All estimates are weighted with analytic weights. Estimates from Model 0 are from Table 3. | | | | | | | | |
